# Subjective Well-Being Under Neuroleptics Scale short form (SWN-K): reliability and validity in an Estonian speaking sample

**DOI:** 10.1186/1744-859X-12-28

**Published:** 2013-09-11

**Authors:** Liina Haring, René Mõttus, Peeter Jaanson, Raine Pilli, Kairi Mägi, Eduard Maron

**Affiliations:** 1 Psychiatry Clinic of Tartu University Hospital, University of Tartu, Tartu, Estonia; 2 Department of Pharmacology, Centre of Excellence for Translational Medicine, University of Tartu, Tartu, Estonia; 3 Centre for Cognitive Ageing and Cognitive Epidemiology, Department of Psychology, University of Edinburgh, Edinburgh, UK; 4 Department of Psychology, University of Tartu, Tartu, Estonia; 5 Jaanson Psychiatric Center, Võru, Estonia; 6 Pärnu Hospital Psychiatric Clinic, Pärnu, Estonia; 7 Marienthal Psychiatry and Psychology Center, Tallinn, Estonia; 8 Division of Brain Sciences, Imperial College London, Faculty of Medicine, London, UK

**Keywords:** SWN scale, Subjective well-being, Psychometric properties, Schizophrenia

## Abstract

**Background:**

The Subjective Well-Being Under Neuroleptic Treatment Scale short form (SWN-K) is a self-rating scale developed to measure mentally ill patients' well-being under the antipsychotic drug treatment. This paper reports on adaptation and psychometric properties of the instrument in an Estonian psychiatric sample.

**Methods:**

In a naturalistic study design, 124 inpatients or outpatients suffering from the first psychotic episode or chronic psychotic illness completed the translated SWN-K instrument. Item content analysis, internal consistency analysis, exploratory principal components analysis, and confirmatory factor analysis were used to construct the Estonian version of the SWN-K (SWN-K-E). Additionally, socio-demographic and clinical data, observer-rated psychopathology, medication side effects, daily antipsychotic drug dosages, and general functioning were assessed at two time points, at baseline and after a 29-week period; the associations of the SWN-K-E scores with these variables were explored.

**Results:**

After having selected 20 items for the Estonian adaptation, the internal consistency of the total SWN-K-E was 0.93 and the subscale consistencies ranged from 0.70 to 0.80. Good test–retest reliabilities were observed for the adapted scale scores, with the correlation of the total score over about 6 months being *r* = 0.70. Confirmatory factor analysis replicated the presence of a higher-order factor (general well-being) and five first-order factors (mental functioning, physical functioning, social integration, emotional regulation, and self-control); the model fitted the data well. The results indicated a moderate-high correlations *r* = 0.54 between the SWN-K-E total score and the evaluation how satisfied patients were with their lives in generally. No significant correlations were found between the overall subjective well-being score and age, severity of the psychopathology, drug adverse effects, or prescribed drug dosage.

**Conclusion:**

Taken together, the results demonstrated that the Estonian version of the SWN-K is a reliable and valid instrument with psychometric properties similar to the original English version. The potential uses of the scale in both research and clinical settings are considered.

## Background

There are substantial individual differences in the manifestation, course, and prognosis among patients diagnosed with a persisting psychotic disorder. In order to reflect such variability among people with psychotic disorders, psychiatrists have broadened the concept of treatment outcome beyond mere symptom improvement by also addressing related phenomena such as subjective well-being or quality of life more generally, management of side effects, subjective response and tolerability to antipsychotics drugs [1]. The present study focuses on the subjective well-being of patients suffering from the psychotic disorder.

The concept of quality of life among the psychiatric patients has an objective and a subjective component. The objective component comprises aspects of “functional status” and “environmental living conditions”, whereas the subjective component refers to perceived “well-being” or “life satisfaction [2].” As for the latter, both illness and treatment can cause distress and contribute to low well-being evaluations. Patients recognize the symptoms of their illness and treatment side effects in similar terms: both can reduce of their well-being. Furthermore, treatment is perceived negatively not only if it fails to sufficiently reduce psychotic symptoms and/or causes substantial side effects but also when it fails to improve well-being for any reason that is not strictly related to the disorder or treatment [3].

General subjective well-being is a broad construct that reflects people's emotional and cognitive evaluations of their lives [4]. The construct is not specific to psychiatric patients: it is a robust dimension of individual and population differences that can be reliably assessed. Just as among psychiatric patients, levels of global well-being vary across healthy individuals [4], cultures [5], and over time [6].

Reported determinants of the self-perceived quality of life or well-being in persons with persisting psychotic disorder include socio-demographic characteristics [7,8], illness-related or clinical characteristics [9,10], psychosocial characteristics [11], and antipsychotic-related side effects [10,12,13]. Additionally, weight gain is prevalent among the patients with schizophrenia and it is associated with poorer quality of life ratings [14].

Nevertheless, there remain some issues pertaining to the assessment of well-being, particularly when the assessments involve different cultures, linguistic groups or psychiatric patient subgroups. According to the previous studies, concerns exist over the psychometric properties of the well-being measures in schizophrenia patients. The first issue is whether to use objective or subjective evaluations. Controversy exists over the reliability of psychotic patients' evaluations of their subjective state. However, objective assessments made by clinicians which have correlated poorly with patients' subjective evaluations of their life quality, are also believed to be inaccurate reflections of patients' well-being [15-17]. For example, studies have found that psychiatrists ignore or tend to minimize patients' complaints about the negative subjective effects of antipsychotic drugs [18] and expectations of antipsychotic treatment differ between patients and physicians [19]. Secondly, the measurement has to be appropriate to the population under study, the clinical condition, and the phase of illness; the instrument has to be adapted to the life of psychotic patients [20] and conform to the context of the culture and value systems in which the patients live [21].

The “Subjective Well-being Under Neuroleptic Treatment” (SWN) scale was created to evaluate the clinical relevance of subjective well-being as a subjective measure of illness and treatment experiences and overall life satisfaction among psychotic disorder patients who vary in illness stage and treatments [12,22,23].

The present study reports the adaptation of the SWN into Estonian. Translation and linguistic validation procedures of the measurements are prerequisite of its use in a new cultural context and only after standardized validation it is possible to compare the research data in a cross-cultural and cross-nation context. The four major aims of the study are as follows: firstly, the study seeks to ensure that the Estonian version of the Subjective Well-Being Under Neuroleptic Treatment Scale short form (SWN-K-E) has the kinds of measurement properties needed to obtain reliable and valid results; secondly, to evaluate the usability of the SWN-K on the patients with the first psychotic episode and on the patients with chronic psychotic disorder. The underlying assumption was that the measurement properties of the subjective well-being scale would be similar for both groups; thirdly, the aim is to evaluate its measurement stability over time and its sensitivity in differentiating between typical or atypical antipsychotic usage. According to the existing literature, we expected that the SWN-K-E scale scores are relatively stable over a period of 6 months, whereas they are to some extent affected by the psychopharmacotherapy (taking into account drug dosage and used first or second generation of antipsychotics). Fourthly, the study assesses the relationship of the Estonian SWN-K-E scores with patients' socio-demographic characteristics, body mass index (BMI), psychopathology, global functioning, and drug adverse effects. These associations (or lack of them) provide information about the validity of the questionnaire but they also address substantive research questions pertaining to the correlates of well-being of psychotic disorder patients. Our expectations were that patients' subjective well-being perceptions would not significantly correlate with psychopathology or drugs side effects. Finally, we assumed that there would be positive correlation between subjective well-being and general functioning because both concepts represent outcomes of the interactions between the patient and the illness, its treatment, as well as social interaction impacts.

## Methods

### Sample characteristics

The combined sample consisted of 124 Estonian-speaking persons, including 68 males (55%) and 56 females (45%) between the ages of 18 and 78 (*M* = 35.7, SD = 13.4 years). A total of 113 participants recompleted the instruments after period of 24 to 56 weeks (*M* = 29.70; SD = 7.66) (response rate 113/124, 91.1%).

The participants with severe physical illness, neurological disorder or learning disability as well as those with acute psychotic symptoms or evidence of organic pathology were excluded. Psychiatric diagnoses were confirmed by examination of medical records to determine the presence of symptoms and illness history. From among the 61 outpatients, 55 met ICD-10 (WHO, 1993) criteria for schizophrenia (F20.08-F29), whereas 6 had schizoaffective disorders (F25); the 63 inpatients were in the early stabilization phase of the first psychotic episode (F23 or F20.09) while they were recruited. The mean age at first hospitalization was 25.6 years (SD = 7.51) for men and 26.07 (SD = 6.94) years for women. The average illness duration for the chronic patients (*n* = 61; 51.6%) was 20.54 (SD = 12.98) years; 63 patients (48.4%) had suffered a first frank psychotic episode and had received antipsychotic treatment for a mean of 3.29 (SD = 2.21) weeks. Patients were treated with various antipsychotic medications as clinically indicated. 99.14% of the patients were receiving antipsychotic medications of either atypical (*n* = 94; 75.80%), typical (*n* = 18; 14.51%) or mixed manner (*n* = 11; 8.87%) and mean theoretical chlorpromazine dose equivalent [24] was 515.08 (SD = 335.03) at the recruitment period, and 443.14 (SD = 299.83) at the follow-up assessment. Eighty eight patients (71%) were treated with only antipsychotics, 10 patients (8%) additionally needed mood stabilizers, 14 (11.3%) and 10 (8.1%) patients respectively received antidepressants or hypnotics in addition to antipsychotic drugs, and 2 patients (1.6%) were on combinations of the afore-mentioned drugs. All participants were in a stable phase when they were recruited; self-evaluated scales addressed the last 7 days. The socio-demographic and clinical characteristics of the participants are presented in Table 1.

**Table 1:** **Socio-demographic characteristics and clinical profile of the sample (*n *= 124)**

**Variables**	**Frequencies, means and SD**		**Frequencies, means and SD**	
	**(Initial sample)**		**(Follow up sample)**	
	**First-episode psychosis patients**	**Chronically ill patients**	**First-episode psychosis patients**	**Chronically ill patients**
	**(*n *= 63)**	**(*n *= 61)**	**(*n *= 58)**	**(*n *= 55)**
Socio-demographic data				
1. Age	27.03 (6.74)	44.80 (12.70)		
2. Sex				
Males	34 (54.0%)	34 (55.7%)	30 (51.7%)	30 (54.5%)
Females	29 (46.0%)	27 (44.3%)	28 (48.3%)	25 (45.5%)
Clinical profile				
1. Treatment duration	23.06 (15.45) days	20.5 (12.98) years		
2. BPRS^a^	23.70 (13.00)	19.05 (8.31)	19.05 (11.00)	16.48 (9.19)
3. GAF^b^	52.78 (11.72)	50.38 (12.20)	59.57 (13.38)	51.93 (12.46)
4. BARS^c^	0.81 (1.79)	1.36 (2.13)	0.55 (1.52)	2.11 (2.53)
5. SAS^c^	0.08 (0.28)	0.25 (0.33)	0.08 (0.22)	0.46 (0.50)
6. AIMS^c^	0.22 (1.31)	2.59 (5.02)	0.15 (0.77)	3.32 (5.92)
7. Chlorpromazine equivalent dosage	387.54 (155.51)	646.81 (412.78)	315.28 (140.04)	568.73 (357.97)
8. Body mass index	23.91 (3.83)	26.07 (4.75)		

^a^Measure of the severity of psychopathology. ^b^Global index of functioning. ^c^Drug adverse effect scales.

### Instruments

#### Subjective well-being scale

The subjective well-being under the neuroleptic treatment scale (SWN-K) [23] assesses patients' perception of their health status, antipsychotic treatment, and nonmedical aspects of their lives. The original version of the SWN is a 38-item scale initially constructed by Naber and coworkers [12] and modified by Naber et al. [23]. This questionnaire is filled out by patients based on their self-perceived symptoms and level of functioning during the preceding 7 days. The SWN short form (SWN-K) consists of 20 statements (10 positive and 10 negative). The SWN has five subscores (mental functioning, self-control, emotional regulation, physical functioning, and social integration), each consisting of four questions. The original SWN and SWN-K (short form) have shown sufficient internal consistency (Cronbach's α was 0.92 for the total score and 0.63 to 0.82 for the subscores, whereas the correlation of the short form total score with the long version was 0.98) [23,25]. It has been reported that the SWN scores are moderately correlated with symptomatology evaluations and its improvement is slightly related to improvement of psychopathology (*r* = −0.20 to −0.37). Therefore, the SWN scale is related to but does not exclusively evaluate symptomatology, i.e., subjective well-being cannot strongly be predicted by means of measuring symptoms of psychotic illness and it is therefore not redundant [23].

#### SWN-K-E translation process

To adapt the SWN short form into Estonian, we followed the Principles of Good Practice for the Translation and Cultural Adaptation Process [26]. The following translation methodology was used: we received permission from the authors who developed the SWN and the SWN short form. Subsequently, two researchers (LH, KK) independently translated the English version of the SWN short form 20-item scale into the target language (Estonian) and three forward-translated versions were generated for the each statement. Divergent results were discussed during consensus meetings with a third researcher (HP). After a linguistic translation, no original item was ruled out as being completely inappropriate for the subsequent cultural adaptation process. The pre-final scale version consisted of sixty (three translations of 20 original items) randomly reordered items. One independent back-translation was performed by the professional translator who was blinded to the original instrument. Items were evaluated by the patients on a Likert-type scale ranging from 1 (strongly disagree) to 6 (strongly agree). The entire instrument is provided in Additional file 1.

#### Satisfaction with life

In addition, participants evaluated an individual item ‘I am generally satisfied with my life’. The responses were given using a five-point Likert-scale, ranging from 1 (strongly disagree) to 5 (strongly agree).

#### Psychopathology

To assess the overall level of psychopathology among the patients, the clinical scale Brief Psychiatric Rating Scale (BPRS) [27] was implemented. The BPRS is a clinician-implemented comprehensive 18-item symptom scale. Each item is measured along a seven-point continuum from “not present” to “extremely severe”.

#### Global functioning

The Global Assessment of Functioning (GAF) is a standard method for measuring patient's overall level of functioning and the severity of psychiatric illness. GAF scores are based on the 100-point scale with descriptions provided for each 10-point interval (higher scores indicate better functioning) [28]. Ratings were based on the clinician's estimate of the patient's current level of functioning in past 7 days.

#### Treatment adverse effects

The Barnes Akathisia Rating Scale (BARS) [29] was used to evaluate drug-induced akathisia. The measurement uses a 5-point scale ranging from 0 to 4. To assess drug-induced abnormal movements, the Abnormal Involuntary Movement Scale (AIMS) [30] was used. The scale contains relevant items, rated on a severity scale of 0 to 4 and patient's awareness of abnormal movements, also rated on a scale of 0 to 4. To assess parkinsonian and related extrapyramidal side effects, the Simpson-Angus Scale (SAS) [31] was implemented. This scale contains 10 items; each item is rated on a Likert scale from 0 to 4. A total score is obtained by averaging item scores.

### Data collection

The research protocol was approved by the Ethic Review Committee on Human Research of the University of Tartu. All participants signed the informed consent after the nature of the procedures had been fully explained. Chart audits were done to collect demographic and clinical data. Patients were enrolled between January 2009 and December 2012. The patients were recruited from Psychiatry Clinic of Tartu University Hospital (*n* = 61); from Tartu, Pärnu and Tallinn Psychiatric Outpatients Centres (*n* = 33) and institutionalized persons from a state nursing home (*n* = 30). The participants were asked to fill a battery of questionnaires and provide standard demographic information about their age, gender, and body mass index. Assessments were carried out at baseline and at 24 to 56 weeks (*M* = 29.70; SD = 7.66) after the first examination.

### Data analysis

Statistical analyses were conducted using the R Statistical software package [32]. Analyses focused on the internal consistency (Cronbach's α) of the SWN-K-E scales, their dimensionality, and construct validity. The principal component analysis (PCA) was conducted in order to replicate the factor structure of the SWN-K-E inventory. An oblique rotation was used to interpret the components because the principal components were inevitably correlated as indicated by the strong first component [33]. In order to achieve the number of items and the structure similar to the original scale, we stepwise-omitted items based on their factor loadings on their perspective factors (items with weakest loadings were dropped). Selected items correlated most strongly with the other items of the subscale and least strongly with other subscales. The number of factors to extract was determined using the eigenvalue greater than 1 criterion (as did the original version of the instrument) and by examining the screeplot. Items with factor loadings ≥0.40 were retained in the scales.

Congruence between the theoretical structures of the SWN-K-E with the structure in the collected data was tested using hierarchical confirmatory factor analysis (CFA) [34]. In the CFA model, each SWN-K-E item (treated as ordered-categorical variable as appropriate) defined its intended factor; no cross-loadings or item residual correlations were allowed. Then, all latent factors were specified to define a general well-being factor. The model was tested using the “lavaan” package [35] and fitted with Diagonally Weighted Least Squares estimator [36]. Model fit indices such as chi-square fit test, the root mean square error of approximation (RMSEA) and the comparative fit index (CFI) were used to evaluate the degree to which the models fitted data. RMSEA values lower than 0.06 and CFI values above 0.95 were considered as evidence for good model fit [37]. Linear multiple regression was used to test associations between SWN-K-E scales and continuous variables, whereas *t* test was employed to examine any group differences in SWN-K-E. Associations with *p* values of less than 0.05 were considered statistically significant. Less than 0.03% of the data were missing; therefore, no imputations were made and pair-wise deletion of missing cases was used where necessary. Evaluated item scores on the SWN-K-E were found to be sufficiently normally distributed.

## Results

### Preliminary analysis

Preliminary, analyses were run to achieve the final 20-item version of the SWN-K-E scale. First, a principal component analysis was conducted on the data obtained on the first assessment. The results indicated that all items loaded on the first unrotated principal component with loadings ranging from 0.27 to 0.74; the first component explained 40.07% of variance in items. According to the PCA, the principal component intercorrelations ranged from *r* = 0.24 to 0.36. Additionally, high communalities (0.56 to 0.81) indicated that the items shared substantial amounts of variance with other items. This suggested there being an overarching well-being dimension. In the next step, five-factor solution was considered, based on the intended structure of the questionnaire.

### Internal consistency

The internal consistency of the total SWN-K-E was 0.93, indicating that selected items showed a reasonable level of shared variance (Table 2). Internal consistencies of the subscales ranged from 0.70 to 0.80, again demonstrating reasonable amounts of shared variance. Item-total correlations ranged from 0.36 to 0.70.

**Table 2:** **Psychometric properties of the SWN-K-E (*n *= 119)**

	**Mean (SD)**	**Cronbach's α**	**Average inter-item correlation**
Total score	84.53 (17.0) [84.79]	0.93 [0.92]	0.37
Mental functioning	16.44 (4.38) [16.24]	0.77 [0.80]	0.49
Social integration	16.55 (4.26) [16.44]	0.80 [0.74]	0.50
Emotional regulation	18.12 (3.92) [17.94]	0.78 [0.73]	0.47
Physical functioning	16.71 (4.04) [16.90]	0.80 [0.82]	0.50
Self control	16.79 (3.81) [17.28]	0.70 [0.63]	0.37

The analogous scores for the original scale [23] are given in brackets.

### Construct validity

**Figure 1:**
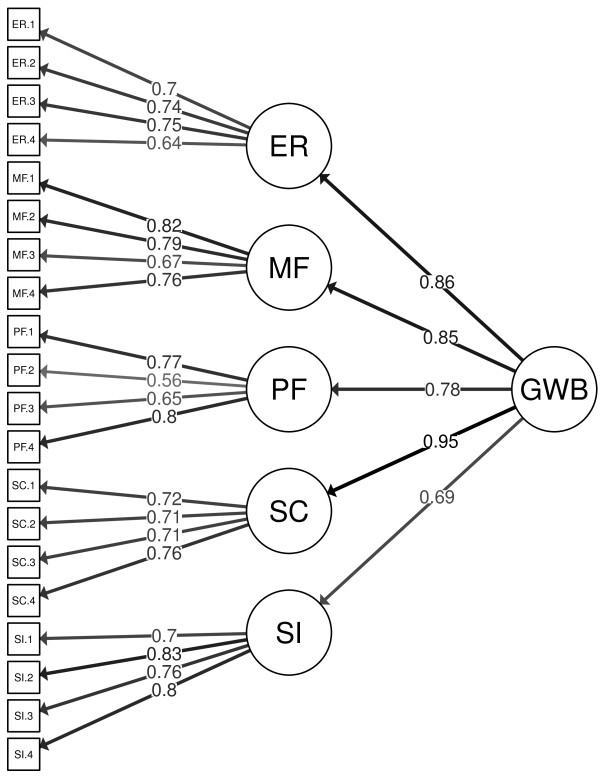
**Parameter estimates for the SWN-K-E scale, according to initial testing (*n *= 120).** GWB, subjective well-being total score; ER, emotional regulation; MF, mental functioning; PF, physical functioning; SC, self-control; SI, social integration; ER.1-SI.4, subscale items, respectively.

CFA was conducted with the selected items of the SWN-K-E in order to replicate the factor structure of the original SWN-K and obtain parameter estimates for a hierarchical solution. The loadings of items on their respective first-order factors varied between 0.56 and 0.83, whereas the loadings of the first-order factors on the general well-being factor ranged from 0.78 to 0.95, respectively (Figure 1).

The specified model fitted the data well (for fit indices, see Table 3).

**Table 3:** **Goodness-of-fit indicators for the SWN-K-E hierarchical factor analysis model (*n *= 120; *n *= 110)**

	**Chi square**	***df***	***p***	**CFI**	**RMSEA**	**CI**
Test	116.156	165	0.999	1.000	0.000	0.000 to 0.000
Retest	129.942	165	0.980	1.000	0.000	0.000 to 0.000

*CFI* comparative fit index, *RMSEA* root mean square error of approximation, CI 90%, confidence interval for RMSEA.

### Test and retest reliability

The adapted instrument was administered twice for the test-retest purposes. Similarly, to the first assessment, the Cronbach's alpha for all items was 0.93 in the retesting, respectively. The data of test-retest reliability can be used to address the issue of instrument stability. Pearson correlation coefficients were established to assess the strength of agreement between the scores at two different times. Subscale correlations between two testing ranged from 0.55 to 0.68 (for ER *r* = 0.63, MF *r* = 0.65, PF *r* = 0.57, SC *r* = 0.55, SI *r* = 0.68, respectively); for total scores, the correlation was 0.70. Therefore, in addition to considerable internal consistency, the SWN-K-E short form showed reasonable temporal stability.

The mean of the total score at the first assessing was 84.53 (SD = 17.00), which did not differ significantly from the second-time mean score (83.34; SD = 17.22; *df* = 103; *t* = 1.00; *p* = ns). Furthermore, the same tendency was seen among four subscales: MF (*M* = 16.44 vs. 15.90; SD = 4.38 vs. 4.11; *df* = 111; *t* = 1.49; *p* = ns); PF (*M* = 16.71 vs. 16.40; SD = 4.04 vs. 4.02; *df* = 109; *t* = 0.18; *p* = ns); SC (*M* = 16.79 vs. 16.99; SD = 3.81 vs. 3.65; *df* = 109; *t* = −0.59; *p* = ns); SI (*M* = 16.55 vs. 16.63; SD = 4.26 vs. 4.04; *df* = 107; *t* = −0.24; *p* = ns). However, for the ER subscore a significant difference occurred (*M* = 18.12 vs. 17.44; SD = 3.92 vs. 4.27; *df* = 110; *t* = 2.13; *p* = 0.04); this may have been due to chance, given the number of tests performed.

A CFA model identical to that described above was fitted to the data from the second measurement and similar estimates were obtained, suggesting that the structural model was also replicable (see Table 3).

### Association of the subjective well-being scores and satisfaction with life

The five SWN-K-E facet scales (MF, PF, SC, SI, and ER) and the total score had moderate to high correlation in the expected direction with how satisfied patients were with their lives in general. Significant correlations ranged from 0.37 to 0.51 for the facets, whereas the correlation was 0.54 for the SWN-K-E total score.

### Subjective well-being index among the first episode vs. chronic psychotic patients

Different patient groups were compared to assess the illness-related factors on the SWN-K-E scores. The comparison was made between patients with first-episode psychosis and patients with chronic psychotic disorder. No significant difference appeared between those two groups in the SWN-K-E total scores (*M* = 86.86 (SD = 16.10) vs. 82.23 (SD = 17.73); *df* = 117; *t* = 1.49; *p* = ns), and the same trend was seen among the facets. The results indicate that the short- or long-term illness or treatment duration do not make a significant contribution to the subjective well-being total score.

### Associations of the SWN-K-E scores with other variables

#### Correlations between the SWN-K-E total score and the demographic characteristics

Results showed no differences between men and women on the SWN-K-E scale total scores: *M* =83.56 vs. 85.62; *df* = 117; *t* = −0.66; *p* = ns and the correlation between the age and SWN-K-E score was non-significant (*r* = −0.06).

#### Impact of the body mass index

No significant correlations were found between BMI and the SWB total index (*r* = −0.12; *p* = ns) but inspection of the SWN-K-E physical functioning subscore and BMI revealed a modest negative correlation (*r* = −0.20; *p* = 0.02).

#### Associations of the SWN-K-E score and antipsychotic treatment

Patients with chronic disease were significantly more often treated with higher antipsychotic drug dosages compared with first-episode psychosis patients. The antipsychotic equivalent dosages were calculated [24] for those two groups at baseline: *M* = 646.81 vs. 387.54; *df* = 122; *t* = 4.66; *p* < 0.01, and during the retesting: *M* = 568.73 vs. 287.94; *df* = 112; *t* = 5.48; *p* < 0.01, respectively. No significant correlations were found between SWN-K-E total scores and using antipsychotics dosage at the baseline (*r* = −0.14), and during the retesting (*r* = −0.04). Furthermore, no significant differences occurred between the SWN-K-E total score and using atypical antipsychotics (*n* = 91) versus typical or combination treatment (*n* = 28) (*M* = 84.98 vs. 83.07; *df* = 117; *t* = 0.51; *p* = ns, at the baseline, and during the retesting: *M* = 84.58 vs. 81.08; *df* = 101; *t* = 0.90; *p* = ns, respectively).

#### Disorder-related dimensions

The patients were characterized by an improvement in general functioning (GAF: *M* = 51.90 vs. 55.88; *df* = 111; *t* = −3.51; *p* < 0.01), and diminishing in severity of illness (BPRS: *M* = 21.23 vs. 17.81; *df* = 111; *t* = 4.0; *p* < 0.01). However, during the follow-up period, extrapyramidal side effects were increased (SAS: *M* = 0.14 vs. 0.21; *df* = 99; *t* = −2.85; *p* < 0.01), and ratings of drug-induced akathisia (BAS), and abnormal movements (AIMS) did not change significantly. Correlations of these variables with the SWN-K-E scores were assessed. A modest positive relationship appeared between the SWN-K-E total scores and the observer-rated global functioning (GAF) scores (*r* = 0.21; *p* < 0.05). No relationships were found between SWN-K-E and psychopathology (BPRS), nor drug side effects (SAS, BAS, and AIMS) scores. These results indicate that the subjective well-being index was not contributed by the demographic variables, BMI, severity of psychopathology, side-effects, and drug dosage, but it was associated with independently rated global functioning.

## Discussion

With an increasing awareness of the variability of treatment outcome and the importance of patient subjective experiences under the treatment, the construct of well-being has become an important area of investigation in psychotic disorder research [38]. Self-report measurements may provide the most direct access to the individual's perceptions of interested domain and therefore, the SWN-K-E scale was adapted as an instrument for research and clinical practice in order to assess the subjective well-being in different dimensions of patients with psychotic disorder treated with antipsychotics.

The process of the instrument development and analysis of validity and reliability results have been rigorously described. Findings provide evidence for adequate internal consistency, as well as for construct validity for the adapted scale. The internal consistency for the adapted scale was found to be the same level with the original scale (Cronbach's *α* = 0.93) [23] and the subscale reliabilities ranged from 0.70 to 0.87. It may therefore be said that the Estonian version of the SWN-K scale is sufficiently internally consistent. In addition, results of the principal component analysis indicated that the SWN-K-E demonstrates the five dimensional factor structure, similar to original scale [23]. The component correlations ranged from *r* = 0.24 to 0.36 suggesting a second-order factor may underlie them. Based on the results, hierarchical confirmatory factor analysis was used to evaluate the structure of the final version of the adapted scale. Five first-order factors (MF, PF, SC, SI, ER) were specified, as per original SWN-K scale [23], and a general higher-order factor. The results of the analysis supported the existence of a higher-order factor (general well-being) and clarified its associations with the first-order factors. The evidence from the current study suggest that, as an alternative factor analytic model, CFA may offer better understanding of the SWN-K factor structure; in particular, it can be used to estimate and parameterize the hierarchical structure of the construct of well-being in psychotic disorder patients.

To evaluate the preliminary convergent validity of the scale, patients were asked to rate how satisfied they generally were with their lives. Moderate correlations (*r* = 0.37 to 0.54) were found between this statement and the SWN-K-E scores. In interpreting these results, it should be considered that single item was used in the comparison, which, due to relatively lower reliability compared to multi-item measures, may have lead to an underestimation of the effect size. In addition, our results showed a weak correlation between SWN-K-E total score and general functioning (GAF). This is in agreement with previous studies [10]. The discriminant validity was examined by looking at the cross-construct correlations which ranged from 0.05 (AIMS) to −0.11 (BPRS). Consistent with previous studies, we found that the patients' subjective well-being was not associated with symptom severity [23] or medication side effects [10]. Our study could not establish the criterion and concurrent validity by reason of lacking of properly adapted existing instruments in the area of health-related quality of life among the psychotic patients in Estonia. There is a need for additional validation studies on the SWN-K-E scale.

Evidence from earlier studies highlight the importance of the assessment of subjective well-being in the course of antipsychotic treatment. The patient satisfaction with antipsychotic therapy seems to correlate strongly to the clinically important aspect of the illness management - patient's adherence to treatment [39]. Moreover, the impact of antipsychotic drugs on subjective well-being has been a controversial issue [10,12]. We did not find a significant association of changes in SWN-K-E total scores and medication type or daily dose of antipsychotic agents. The fact that no differences with regards to SWN between people under different antipsychotic treatment were detected in this sample should be interpreted with caution. Due to the small sample size in the typical antipsychotic treatment subgroup, there was not enough statistical power to exclude false-negative results. Moreover, we did not implement controlled drug change intervention and the patients were not taking only antipsychotics during the study period. Stability of the measurement versus sensitivity to change is an important issue for the adapted instrument. Therefore, whether a high degree of stability is encouraging or discouraging for the considered interpretation depends upon the theory defining the construct [40]. Our study provided evidence for the average stability of well-being judgments (retest reliability).

It has been demonstrated that most psychiatric patients are reliably and consistently able to express their inner feelings and their level of satisfaction [41,42]. However, when employing self-evaluating scales in psychiatric patient groups, we should acknowledge that the lack of insight into their illness, affective and cognitive symptoms or reality distortion may imply difficulties in understanding the items of the scale [43], which may impair scale validity.

The subjects included in this study were symptomatic but stable psychotic patients, maintained on regular antipsychotic therapy. Such a profile closely resembles the type of patients seen in psychiatric clinics. Regardless, the serious mental illness the majority of participating patients seemed to be quite satisfied with their life.

### Limitations

The results of this study should be interpreted with caution because of several methodological limitations. Firstly, this work was limited with respect to its sample, foremost in terms of size. Our study provides preliminary findings; further research examining individuals diagnosed with psychotic illness with varying clinical presentations and under the different treatment settings is warranted in order to delineate the potential predictors of well-being in this clinical group. Secondly, the heterogeneity of our study sample may create important generalizability problems. We cannot be sure to what extent these findings can be extended to other specific subgroups of psychotic patients, especially those with severe cognitive impairment and judgement difficulties. Thirdly, the extent of well-being and its determinants need to be investigated more longitudinally, to study subtle changes throughout the course of mental illness. For further studies, focusing on different groups in terms of age, more detailed social characteristics, and how different cognitive dysfunction domains affect the well-being judgements are recommended. Fourthly, further research is needed to evaluate the criterion validity of the SWN-K-E.

Nonetheless, this work provides support for the use of SWN-K-E to measure the subjective well-being construct of the psychotic patients in research and clinical purposes.

## Conclusions

Taken together, we provided some evidence that adapted SWB-K-E scale is a reliable and valid instrument for measuring the subjective well-being among the patients who are suffering from psychotic illness. Our study identified the theoretical structure of the adapted SWN-K scale and suggests that the total score and subscores of the scale could be used as indicators for the total or specific domain of the construct.

Clinicians should become more aware of the subjectively distressing nature of the psychotic illness as well as the impact of the antipsychotic drug therapy on the well-being, and take into consideration the patients' evaluations while managing multidimensional approach to the treatment. The assessment of well-being is not redundant and information on their well-being may further help to improve the quality of life care of people with mental illness.

## Abbreviations

SWN: Subjective well-being under neuroleptics scale; SWN-K: Subjective well-being under neuroleptics scale short form; SWN-K-E: Subjective well-being under neuroleptics scale short form, Estonian version; BPRS: Brief psychiatric rating scale; GAF: Global assessment of functioning; BARS: Barnes akathisia rating scale; AIMS: Abnormal involuntary movement scale; SAS: Simpson-Angus scale; BMI: Body mass index; PC: Principal component; PCA: Principal component analysis; CFA: Confirmatory factor analysis; RMSEA: Root mean square error of approximation; CFI: Comparative fit index.

## Competing interests

The authors report no conflicts of interest. The authors alone are responsible for the content and writing of the paper.

## Authors’ contributions

LH was responsible for the study conception and design, participated in the study organization and data collecting, as well as writing the manuscript. RM and LH performed the statistical analysis; PJ, RP, and KM performed clinical assessments, and RM and EM made critical revisions to the paper for important intellectual content. All authors read and approved the final manuscript.

## Supplementary Material

Additional file 1 SWN-K and adapted SWN-K-E items. Click here for file

## References

[B1] Voruganti LNP Awad AG Personal Evaluation of Transitions in Treatment (PETiT): A scale to measure subjective aspects of antipsychotic drug therapy in schizophrenia Schizophr Res 2002 56 1–2 37 46 12084418 10.1016/s0920-9964(01)00161-x

[B2] Ritsner MS Awad AG Quality of life impairment in schizophrenia, mood and anxiety disorders: New perspectives on research and treatment 2007 New York, NY US: Springer Science + Business Media

[B3] Carrick R Mitchell A Powell RA Lloyd K The quest for well-being: a qualitative study of the experience of taking antipsychotic medication Psychol Psychother 2004 77 19 33 10.1348/147608304322874236 15025902

[B4] Diener E Oishi S Lucas RE Personality, culture, and subjective well-being: emotional and cognitive evaluations of life Annu Rev Psychol 2003 54 403 425 10.1146/annurev.psych.54.101601.145056 12172000

[B5] Schimmack U Radhakrishnan P Oishi S Dzokoto V Ahadi S Culture, personality, and subjective well-being: integrating process models of life satisfaction J Pers Soc Psychol 2002 82 4 582 593 11999925

[B6] Schimmack U Oishi S The influence of chronically and temporarily accessible information on life satisfaction judgments J Pers Soc Psychol 2005 89 3 395 406 16248721 10.1037/0022-3514.89.3.395

[B7] Caron J Mercier C Diaz P Martin A Socio-demographic and clinical predictors of quality of life in patients with schizophrenia or schizo-affective disorder Psychiatry Res 2005 137 3 203 213 10.1016/j.psychres.2005.07.002 16298428

[B8] Fujimaki K Morinobu S Yamashita H Takahashi T Yamawaki S Predictors of quality of life in inpatients with schizophrenia Psychiatry Res 2012 197 3 199 205 10.1016/j.psychres.2011.10.023 22370148

[B9] Eack SM Newhill CE Anderson CM Rotondi AJ Quality of life for persons living with schizophrenia: more than just symptoms Psychiatr Rehabil J 2007 30 3 219 222 17269273 10.2975/30.3.2007.219.222 PMC3716361

[B10] Ritsner MS Lisker A Arbitman M Ten-year quality of life outcomes among patients with schizophrenia and schizoaffective disorders: I. Predictive value of disorder-related factors Qual Life Res 2012 21 5 837 847 10.1007/s11136-011-9988-2 21912845

[B11] Ritsner MS Arbitman M Lisker A Ponizovsky AM Ten-year quality of life outcomes among patients with schizophrenia and schizoaffective disorder II. Predictive value of psychosocial factors Qual Life Res 2012 21 6 1075 1084 10.1007/s11136-011-0015-4 21964946

[B12] Naber D A self-rating to measure subjective effects of neuroleptic drugs, relationships to objective psychopathology, quality of life, compliance and other clinical variables Int Clin Psychopharmacol 1995 10 Suppl 3 133 138 8866775

[B13] Schimmelmann BG Moritz S Karow A Schafer I Bussopulos A Golks D Krausz M Naber D Lambert M Correlates of subjective well-being in schizophrenic patients treated with atypical antipsychotics Int J Psychiatry Clin Pract 2005 9 2 94 98 10.1080/13651500510018266 24930789

[B14] Allison DB Mackell JA McDonnell DD The impact of weight gain on quality of life among persons with schizophrenia Psychiatr Serv 2003 54 4 565 567 10.1176/appi.ps.54.4.565 12663847

[B15] Lehman AF Postrado LT Rachuba LT Convergent validation of quality of life assessments for persons with severe mental illnesses Qual Life Res 1993 2 5 327 333 10.1007/BF00449427 8136797

[B16] Sainfort F Becker M Diamond R Judgments of quality of life of individuals with severe mental disorders: patient self-report versus provider perspectives Am J Psychiatry 1996 153 4 497 502 8599397 10.1176/ajp.153.4.497

[B17] Voruganti L Cortese L Oyewumi L Cernovsky Z Zirul S Awad A Comparative evaluation of conventional and novel antipsychotic drugs with reference to their subjective tolerability, side-effect profile and impact on quality of life Schizophr Res 2000 43 2–3 135 145 10858632 10.1016/s0920-9964(99)00154-1

[B18] Seale C Chaplin R Lelliott P Quirk A Antipsychotic medication, sedation and mental clouding: an observational study of psychiatric consultations Soc Sci Med 2007 65 4 698 711 10.1016/j.socscimed.2007.03.047 17507129

[B19] Chue P The relationship between patient satisfaction and treatment outcomes in schizophrenia J Psychopharmacol 2006 20 6 38 56 17046986 10.1177/1359786806071246

[B20] Awad AG Voruganti LNP Intervention research in psychosis: Issues related to the assessment of quality of life Schizophr Bull 2000 26 3 557 564 10.1093/oxfordjournals.schbul.a033477 10993397

[B21] Skevington SM Advancing cross-cultural research on quality of life: observations drawn from the WHOQOL development. World Health Organisation Quality of Life Assessment Qual Life Res 2002 11 2 135 144 10.1023/A:1015013312456 12018737

[B22] de Haan L Nimwegen L Amelsvoort T Dingemans P Linszen D Improvement of subjective well-being and enduring symptomatic remission, a 5-year follow-up of first episode schizophrenia Pharmacopsychiatry 2008 41 4 125 128 10.1055/s-2008-1076729 18651339

[B23] Naber D Moritz S Lambert M Pajonk FG Holzbach R Mass R Andresen B Improvement of schizophrenic patients' subjective well-being under atypical antipsychotic drugs Schizophr Res 2001 50 1–2 79 88 11378316 10.1016/s0920-9964(00)00166-3

[B24] Gardner DM Murphy AL O'Donnell H Centorrino F Baldessarini RJ International consensus study of antipsychotic dosing Am J Psychiatry 2010 167 6 686 693 10.1176/appi.ajp.2009.09060802 20360319

[B25] Schmidt P Clouth J Haggenmüller L Naber D Reitberger U Constructing an Index for the Subjective Well-being Under Neuroleptics scale (SWN), short form: applying structural equation modeling for testing reliability and validity of the index Qual Life Res 2006 15 7 1191 1202 10.1007/s11136-006-0069-x 17004003

[B26] Wild D Grove A Martin M Eremenco S McElroy S Verjee-Lorenz A Erikson P Principles of good practice for the translation and cultural adaptation process for Patient-Reported Outcomes (PRO) measures: report of the ISPOR task force for translation and cultural adaptation Value Health 2005 8 2 94 104 10.1111/j.1524-4733.2005.04054.x 15804318

[B27] Overall JE Gorham DR The brief psychiatric rating scale Psychol Rep 1962 10 3 799 812 10.2466/pr0.1962.10.3.799

[B28] Endicott J Spitzer RL Fleiss JL Cohen J The global assessment scale: a procedure for measuring overall severity of psychiatric disturbance Arch Gen Psychiatry 1976 33 6 766 771 10.1001/archpsyc.1976.01770060086012 938196

[B29] Barnes TR A rating scale for drug-induced akathisia Br J Psychiatry 1989 154 672 676 10.1192/bjp.154.5.672 2574607

[B30] Guy WA Abnormal Involuntary Movement Scale (AIMS) 1976 Washington, DC: ECDEU Assessment Manual for Psychopharmacology, U.S. Department of Health Education and Welfare 534 537

[B31] Simpson GM Angus JWS A rating scale for extrapyramidal side effects Acta Psychiatr Scand 1970 212 11 19 10.1111/j.1600-0447.1970.tb02066.x 4917967

[B32] R Development Core Team. R A language and environment for statistical computing 2012 Vienna, Austria: R Foundation for Statistical Computing http://www.R-project.org/

[B33] Fabrigar LR Wegener DT MacCallum RC Strahan EJ Evaluating the use of exploratory factor analysis in psychological research Psychol Methods 1999 4 3 272 299

[B34] Schmid J Leiman JM The development of hierarchical factor solutions Psychometrika 1957 22 53 61 10.1007/BF02289209

[B35] Rosseel Y Iavaan: an R package for structural equation modeling J Stat Softw 2012 48 2 1 36

[B36] Christoffersson A Two-step weighted least squares factor analysis of dichotomized variables Psychometrika 1977 42 3 433 438 10.1007/BF02293660

[B37] Hu L Bentler PM Cutoff criteria for fit indexes in covariance structure analysis: conventional criteria versus new alternatives Struct Equ Model 1999 6 1 1 55 10.1080/10705519909540118

[B38] Vothknecht S Schoevers RA de Haan L Subjective well-being in schizophrenia as measured with the Subjective Well-Being under Neuroleptic Treatment scale: a review Aust N Z J Psychiatry 2011 45 3 182 192 10.3109/00048674.2010.545984 21438745

[B39] Naber D Karow A Lambert M Subjective well-being under the neuroleptic treatment and its relevance for compliance Acta Psychiatr Scand Suppl 2005 427 29 34 15943008 10.1111/j.1600-0447.2005.00542.x

[B40] Cronbach LJ Meehl PE Constructs validity in psychological tests Psychol Bull 1955 52 281 302 13245896 10.1037/h0040957

[B41] Awad AG Voruganti LN Heslegrave RJ Measuring quality of life in patients with schizophrenia Pharmacoeconomics 1997 11 1 32 47 10.2165/00019053-199711010-00005 10172917

[B42] Skantze K Malm U Dencker SJ May PR Comparison of quality of life with standard of living in schizophrenic out-patients Br J Psychiatry 1992 161 797 801 10.1192/bjp.161.6.797 1483165

[B43] Katschnig H Katschnig H, Freeman H, Sartorius N How Useful Is the Concept of Quality of Life in Psychiatry? 2006 New York, NY: Wiley 3 17

