# Supplementary material for: Subjective Well-Being Under Neuroleptics Scale short form (SWN-K): reliability and validity in an Estonian speaking sample
Source: Ann Gen Psychiatry. 2013 Sep 11;12:28. doi: 10.1186/1744-859X-12-28 (PMC3847444; doi:10.1186/1744-859X-12-28)
Supplement: Additional file 1 — SWN-K and adapted SWN-K-E items. [file 1744-859X-12-28-S1.doc]

**Additional file 1**

**SWN-K and adapted SWN-K-E items**

| **Item designation** | **SWN-K item** | ***SWN-K-E item*** |
| --- | --- | --- |
| SC.2 | I feel powerless and not in control of myself. | *Mul on keeruline enda üle kontrolli säilitada.* |
| PF.2 | I feel very comfortable with my body. | *Tunnen end oma kehas väga hästi.* |
| MF.2 | I find it easy to think. | *Mõtlemine ei valmista mulle probleeme.* |
| ER.2 | I have no hope for the future. | *Tulevik tundub mulle lootusetu.* |
| PF.3 | My body feels familiar. | *Olen oma keha suhtes salliv.* |
| SI.1 | I am very shy about getting to know people. | *Olen inimestega tutvumisel väga häbelik.* |
| MF.3 | I am imaginative and full of ideas. | *Olen loov ja ideedest tulvil.* |
| SI.3 | My environment seems friendly and familiar to me. | *Mind ümbritsev keskkond on sõbralik ja turvaline.* |
| PF.4 | I feel weak and exhausted. | *Mind vaevab nõrkus ja kurnatus.* |
| ER.3 | My emotions and sensations are dull. Nothing matters me. | *Olen emotsionaalselt tuim, miski pole mulle oluline.* |
| MF.9 | My thinking is difficult and slow. | *Mõtlemine valmistab mulle raskusi ja nõuab aega.* |
| SC.5 | My feelings and behaviours are inappropriate to situations. I get upset over small things, important ones hardly affect me. | *Minu tunded ja käitumine ei ole kooskõlas: tühised asjad häirivad mind ja samas jätavad olulised teemad külmaks.* |
| SI.9 | I find it easy to keep in touch with people around me. | *Mul on kerge suhelda teiste inimestega.* |
| SI.2 | I perceive my environment as being changed, strange and threatening. | *Minu arvates muutub mind ümbritsev keskkond üha võõramaks ja ähvardavamaks.* |
| SC.9 | I find it easy to draw a line between myself and others. | *Mul on kerge eristada end teistest.* |
| PF.1 | My body is a burden to me. | *Mu keha tekitab minus vastumeelsust.* |
| MF.1 | My thoughts are flighty and undirected. I find it difficult to think clearly. | *Mul on raske oma mõtteid koondada ja eesmärgipäraselt mõelda.* |
| ER.4 | I am interested in what is happening around me, and it is important to me. | *Minu ümber toimuv pakub mulle huvi ja on mulle oluline.* |
| SC.1 | My feelings and behaviour are appropriate in the particular situation. | *Mu tunded ja käitumine sobivad situatsioonidega kokku.* |
| ER.1 | I am full of confidence, everything will be alright. | *Ma olen eneses kindel ja veendunud, et kõik läheb hästi.* |
